# Many different roads lead to Rome: equivalence of time-use for activity, sedentary and sleep behaviours and dietary intake profiles among adolescents

**DOI:** 10.1186/s44167-022-00005-1

**Published:** 2022-11-01

**Authors:** Dorothea Dumuid, Maddison L. Mellow, Tyman E. Stanford, Kar Hau Chong, Susan M. Sawyer, Ashleigh E. Smith, Charlotte Lund Rasmussen, Alexandra Wade, Timothy Olds

**Affiliations:** 1 Alliance for Research in Exercise, Nutrition and Activity, Allied Health & Human Performance, University of South Australia, City East Campus, Frome Rd, GPO Box 2471, Adelaide, SA 5001 Australia; 2 Centre for Adolescent Health, Royal Children’s Hospital and Murdoch Children’s Research Institute, The University of Melbourne, Parkville, VIC Australia; 3 Department of Paediatrics, The University of Melbourne, VIC Parkville, Australia; 4 Early Start, School of Health and Society, Faculty of the Arts, Social Sciences and Humanities, University of Wollongong, Wollongong, NSW Australia; 5 Department of Public Health and Nursing, Norwegian University of Science and Technology, Trondheim, Norway; 6 Faculty of Physical Culture, Palacký University, Olomouc, Czech Republic

**Keywords:** Time use, Diet, Health, Adolescent, Non-communicable diseases

## Abstract

**Background:**

How we spend our time and what we eat have important implications for our health. Evidence suggests that health-equivalent behaviour change options which result in the same benefit are available within both time use (physical activities, sedentary behaviours and sleep) and diet (e.g., fruit and vegetables, snack foods). However, it is not yet known if health-equivalent choices exist across both time-use and diet behaviours. This study aimed to explore if a variety of different time-use and dietary profiles were associated with equivalent physical functioning score among adolescents.

**Methods:**

This study used cross-sectional data from 2123 adolescent participants from the Longitudinal Study of Australian Children (LSAC) (mean age = 14.4 ± 0.5 years), including time-use diaries (min/day of sleep, self-care, screen time, quiet time, physical activity, school-related and domestic/social), diet questionnaires (serves/day of fruit and vegetables, discretionary (snack) foods and sugar-sweetened beverages) and a measure of physical functioning (PedsQL™ 4.0 physical functioning scale for teens). Multiple linear regression models were used to find the association of 24-h time-use composition (expressed as isometric log ratios) and dietary variables with physical functioning score. The models were used to estimate which time-use and diet profiles (within a feasible range from the sample average) were associated with equivalent physical functioning scores. Finally, an interactive app was developed to make the results accessible to end users.

**Results:**

Within 30 min and 1.5 servings of the average adolescent’s time-use and dietary behaviours, 45 equivalent options were associated with a ~ 0.2 SD improvement in physical functioning scale. All options associated with this improvement in physical function involved increasing physical activity and increasing fruit and vegetable intake, whilst also reducing discretionary food intake and sugar-sweetened beverages. Most behavioural options also increased sleep and reduced time spent in self-care, screen time and quiet time activities.

**Conclusions:**

There are a range of time-use and diet profiles that may result in equivalent benefits in physical functioning among adolescents. Communicating these options using decision tools such as interactive apps may allow for tailored interventions across both time use and diet which are based on an individual’s needs, preferences and constraints.

**Supplementary Information:**

The online version contains supplementary material available at 10.1186/s44167-022-00005-1.

## Background

Both what we do with our time and what we eat have important links with health outcomes as diverse as depression, blood pressure, cognition and bone strength [1–4]. It is very likely that the same improvement in health could be achieved in different ways. If different time reallocations or dietary adjustments have equivalent effects on health, people could have greater flexibility and choice as to which lifestyle change best suits them. This would have profound implications for lifestyle interventions as instead of “one-size-fits-all” recommendations, interventions could be tailored to an individual’s preferences, needs and constraints. Providing an individual with ownership and autonomy may be the key to facilitating behaviour change that is sustainable beyond specific interventions [5].

Time-equivalency has been recently explored in relation to health measures. For adiposity among school-aged children, Dumuid et al. [6] found that an equivalent reduction in body mass index z-score (zBMI) of 0.1 was associated with either 48 min/d more sleep, 108 min less sedentary time, 21 min less light physical activity or 8 min more moderate-to-vigorous physical activity (MVPA) (all considered relative to the remaining behaviours, and relative to the sample compositional mean). Talarico and Janssen [7] reported similar findings among a sample of children. For other health measures, Ng et al. [8] found that, in relation to a 0.1 standardised increase in health-related quality of life, equivalent options included 64 min more sleep, 65 min less sedentary time, 72 min less light physical activity or 29 min more moderate-to-vigorous physical activity (each relative to the remaining behaviours, and to the sample compositional mean). They also reported that a 0.1 standardised increase in academic achievement was associated with 95 min more of sedentary time or 52 min less of light physical activity. Among adults, Chastin et al. [9] reported equivalent associations with all-cause mortality for two very different daily activity profiles: 3 min moderate-to-vigorous physical activity, 375 min light physical activity, 582 min sedentary time, and 480 min sleep or 55 min moderate-to-vigorous physical activity, 250 min light physical activity, 655 min sedentary time, and 480 min sleep. While in all of these analyses, MVPA is by far the strongest predictor on a minute-for-minute basis, it is clear that many different trade-offs are associated with equivalent outcomes.

Less attention has been given to the equivalence of different diet profiles on health outcomes. *Nutritional geometry* is a framework that encourages diet and health research to move away from single nutrients or dietary components and instead consider the whole diet in relation to health outcomes [10]. Using this approach, which has been mostly applied to animal research to date, Solon-Biet et al. [11] demonstrated that mice (fed ad libitum) on low protein, high carbohydrate diets had better metabolic health and increased longevity compared to mice on high protein, low carbohydrate or low protein, high fat diets across a range of energy intake profiles. Similarly, Lee et al. [12] demonstrated that caloric intake was not related to longevity in *Drosophila* (flies), but rather the ratio of protein to carbohydrates within the diet (regardless of energy intake) had the strongest relationship with longevity. Together, these studies acknowledge that dietary changes such as caloric restriction may influence health outcomes not only by the change in energy intake, but through the re-allocation of macronutrient intake. Several nutritional epidemiology studies have assessed the effects of *isocaloric substitution* on health outcomes in human populations. Isocaloric substitution models explore the effects of replacing one macronutrient with another while keeping the remaining macronutrient intakes constant [13]. Applying this approach to human data, Melaku et al. [14] found that substituting 5% of energy intake from protein for saturated fat or carbohydrate increased the odds of excessive daytime sleepiness. The same effects were also found when carbohydrate was substituted with saturated fat [14]. These findings support the potential for a range of reallocations between diet components which could lead to equivalent health outcomes. These past studies have concentrated on macronutrient substitutions, but studies considering the health associations of swaps between food groups (e.g., fruit and vegetables, discretionary foods) may be more meaningful to everyday people, and more useful for research translation.

Dietary intake interacts with time-use behaviour to influence health [15], and several behaviour changes across diet and time-use profiles could result in the same health outcome. Although providing individuals with the autonomy to select the most suitable health behaviour changes holds many benefits, it also presents the individual with a series of decisions that need to be made. To reduce the complexity of these decisions across multiple health behaviours (i.e. across the time-use and diet profile), decision tools are needed to ensure optimal understanding of required changes and their benefits for health [16]. Indeed, a significant challenge in health research is ensuring that findings are translated and disseminated in a way that facilitates behaviour change beyond the end of intervention periods (i.e., health behaviours are adopted in real-life settings) [5]. Decision tools which guide an individual through the various health behaviour changes that could achieve the same effect (i.e. across both time-use and diet options) would facilitate autonomy and understanding, and help ensure that the behaviour changes are suited to the individual and their needs, constraints and preferences.

Although time-use and diet behaviours are associated with many of the same health outcomes, to our knowledge no one has explored if equivalent behavioural mixes exist across both time use and diet. This study aimed to explore the equivalency of time-use and dietary profiles in relation to a holistic measure of physical functioning using adolescent data from a large, population-based study. We then set out to develop an interactive app that could be used to translate the findings.

## Methods

The Longitudinal Study of Australian Children (LSAC) [17] recruited two nationally-representative cohorts in 2003/4 using a two-staged clustered sampling strategy; a birth (B) cohort (n = 5107) and a child (K) cohort (n = 4983). There were no exclusion criteria. Ethical approval for the LSAC study was obtained from the Australian Institute of Family Studies Ethics Committee and informed consent was obtained from parents/guardians of all participants. The participants are followed up in biennial waves. This study uses cross-sectional data from Wave 6 (2013/4) of LSAC’s K cohort, when the participants were 14 years old.

### Time-use data (independent variable)

Participants were mailed an open-ended paper diary and were instructed to complete it on the day before their scheduled home interview [18]. Participants recorded the time (hour and minutes) they woke up, when they went to bed and when they went to sleep. In addition, they recorded the start time of each activity they did during the day. At the home visit, the interviewer took the participant through the diary, prompting them for further information to fill in any gaps. The interviewer transposed the recorded activities using a predetermined coding framework to create comparable activity types across all participants’ diaries. The time-use diaries were processed following a standardised protocol to detect invalid data (Additional file 1). Daily time spent in each activity was calculated as the difference between the start time of the activity and the start time of the next activity. As described in the Additional file 1, activities were aggregated into seven time-use behaviours: Sleep, Self-Care, Screen Time, School-Related, Quiet Time, Physical Activity and Domestic/Social Activities. The seven time-use behaviours were checked for any zero values, which would preclude the log-ratio transformation required for compositional data analysis. There were zeros in all behaviours except Sleep and Self Care (see Additional file 1 for details). Because it is reasonable to assume that, if time use was measured over a long enough period or using a more precise instrument, all adolescents would eventually accumulate some time in each of the seven time-use behaviours, zeros were replaced with small values using the log-ratio expectation–maximisation algorithm in the “robCompositions” package [19]. The log-ratio expectation maximisation algorithm determines the optimal small value to use to replace the zero, with the aim of minimizing the disruption to the data’s relative covariance structure. The maximum replacement value was set at 10 min. Each participant recorded 1 diary day. Of the days collected, 64% were school days.

### Diet data (independent variable)

Participants self-reported dietary intake using paper-based questionnaires. Responses were coded as: 0 = “Not at all”; 1 = “Once”; 2 = “Twice”; and 3 = “More than twice” for each of the questions below. For this study, we considered each instance of intake to be equivalent to one serving. Thus the first three questions were summed to create a daily “Fruit and Vegetables” serving score (0–9): “Thinking about yesterday, how often did you have … fresh fruit?”; “…cooked vegetables?”; “…raw vegetables and salad?”. The next four questions were summed to create a daily “Discretionary Food” serving score (0–12): “Thinking about yesterday, how often did you have … meat pie, hamburger, hot dog, sausage or sausage roll?”; “… hot chips or French fries?”; “… potato chips or savoury snacks such as ‘Twisties’?” “… biscuits, doughnuts, cake or chocolate?”. The following two questions were summed to create a daily “Sugar-Sweetened Beverages” serving score (0–6): “Thinking about yesterday, how often did you have …energy drinks (e.g. Redbull, Mother or V)?”; “… soft drink or cordial (not diet soft drink or diet cordial)?”.

### Physical functioning (dependent variable)

Participants completed the PedsQL™ 4.0 physical functioning scale for teens (13–18 years) [20, 21]. They selected a response from: 0 = “Never”; 1 = “Almost never”; 2 = “Sometimes”; 3 = “Often”; 4 = “Almost always”, for eight questions about their physical functioning. Questions included: “In the last month, how much of a problem has this been for you?… (1) “It is difficult for me to walk more than 100 m”; (2) “It is difficult for me to run”; (3) “It is difficult for me to play sport or do exercise”; (4) “It is difficult for me to lift something heavy”; (5) “It is difficult for me to have a bath or shower by myself”; (6) “It is difficult for me to help around the house”; (7) “I get aches and pains”; (8) “I have low energy”. The mean of the responses was used (if less than five items were missing, otherwise score was classified as missing), and scaled to be between 0 and 100, where a higher score indicates better physical functioning.

### Covariates

Age in years and sex were gathered in the LSAC parent survey. Family-level socioeconomic position was a composite z-score created for LSAC incorporating the family’s combined income, parental occupation and education, which were reported in the parent survey [22]. These variables were considered as confounders due to their potential influence on time use, diet and physical functioning.

### Statistical analysis

Analyses were performed in R Version 4.1.0 (R Core Team, Vienna, Austria), using the “Compositions” package [23]. The R “Shiny” package [24] was used to create the interactive online interface.

The relationship of time-use behaviours and dietary intake with physical functioning was explored using a multiple linear regression model. The seven time-use behaviours were conceptualised as a 24-h time-use composition [25]. To deal with the perfect multi-collinearity between the time-use behaviours, and enable their inclusion in the linear model, they were expressed as a set of six isometric log ratios [6] using the *ilr()* function from the “Compositions” package. Interactions between sex and the behavioural variables were tested and all found to be statistically non-significant (all p > 0.30), indicating that stratification by sex was not warranted. Sex, age and household socioeconomic position were included as covariates. To normalise the model’s residuals, a BoxCox transformation [26] was applied to the physical functioning scale. The multivariate F statistic from the ANOVA table of the above model fit was used to determine whether the overall time-use composition was associated with physical functioning, while standardised betas (std_beta) from the multiple linear regression model were reported for each of the dietary intake variables.

An iterative approach was used to determine equivalences of time-use behaviours and dietary intake. The multiple linear regression model described above was used as a predictive formula to estimate physical functioning for systematically created time-reallocations and dietary choices [16]. Starting from the sample average (time-use compositional mean rounded to nearest 10 min) an equally spaced grid was created, simulating all possible time reallocations from − 30 to + 30 min in 10-min increments. To keep the simulated scenarios within feasible bounds, reallocations were restricted within a 30-min radius from the average time-use composition. Similarly, simulated diet servings ranged from − 1.5 (− 1 for sugar-sweetened beverages to avoid negative values) to + 1.5 servings from the average serve (rounded to the nearest integer), in 0.5-serve increments. The physical functioning scale score was estimated for all possible combinations of time-use and dietary intake profiles using the multiple linear regression model [27]. Covariates were kept constant at their mean/mode. All time use and dietary profiles associated with a positive difference of 0.5, 1, 1.5, 2 and 2.5 in physical functioning scale were extracted and plotted in stacked bar plots to enable visualisation of trends.

A ShinyApp interface was created to enable the user to interrogate potential time-use and dietary equivalences for their own selected physical functioning differences. The app was designed to provide the user the choice of which time-use or diet behaviour they would like to change first, and by how much. Subsequently, the user is prompted to select their next behaviour choice, until all behaviours have been accounted for.

## Results

Of 3537K-cohort participants in Wave 6, 3074 provided a time-use diary, of which 2166 were considered valid (Additional file 1). A further 2146 participants had complete dietary data, and 2138 also had complete physical functioning data. The final analytical sample consisted of 2123 adolescent participants with complete covariates, outcome and exposure data (Table 1).

**Table 1:** Characteristics of sample

Characteristic	N = 2123
Sex, n (%) female	1064 (50)
Age, years mean (SD)	14.4 (0.5)
SEP, z-score mean (SD)	0.06 (1.00)
Time-use behaviours, min/day, arithmetic mean (SD)	
Sleep	553 (82)
Self-care	107 (59)
Screen time	203 (169)
Quiet time	170 (116)
Physical activity	87 (100)
School-related	227 (192)
Domestic/social	94 (109)
Time-use behaviours, min/day, compositional mean	
Sleep	741
Self-care	125
Screen time	163
Quiet time	175
Physical activity	50
School-related	129
Domestic/social	58
Dietary intake, servings/day, mean (SD)	
Fruit and vegetables	3.7 (2.2)
Discretionary foods	2.0 (1.7)
Sugar-sweetened beverages	0.6 (1.0)
PedsQL™ 4.0 teen physical functioning scale	89.3 (12.8)

SEP: socioeconomic position; PedsQL™: pediatric quality of life

The overall time-use composition was associated with physical functioning (F = 4.3, p < 0.001), as was intake of fruit and vegetables (std_beta = 0.05, p < 0.001), discretionary foods (std_beta = − 0.04, p = 0.008) and sugar-sweetened beverages (std_beta = − 0.06, p = 0.01).

**Fig. 1:**
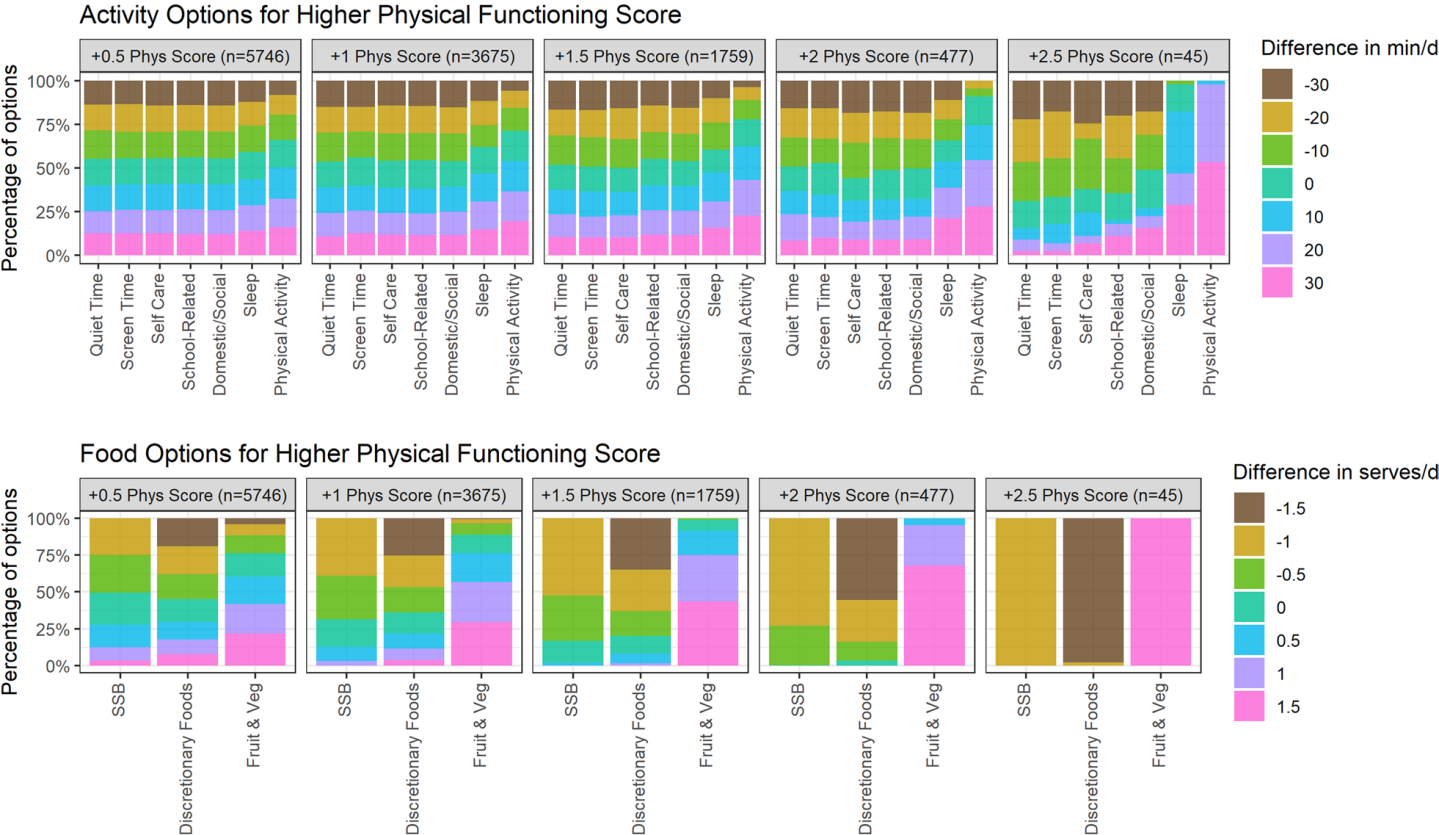
Simulated equivalent behaviour-change options for physical functioning across time use and dietary intake. For each time-use behaviour (displayed in top five cells) and dietary choice (displayed in bottom five cells), the percentage of options associated with increases in physical functioning of + 0.5, + 1, + 1.5, + 2 or + 2.5 points is shown. For example, the top-most right cell displays what percent of options for changes in each time-use behaviour are associated with + 2.5 improvement in physical functioning, showing that there are no options to reduce physical activity, but that the majority of options require reductions in quiet time, screen time and self-care (brown/green colours). In terms of diet, the only options available for a + 2.5 improvement in physical functioning are increases in fruit and vegetables, and decreases in SSBs and discretionary foods. Models were adjusted for age, sex and household socioeconomic position. Reference durations (zero difference) for time-use behaviours are: sleep = 740; self care = 130; screen time = 160; quiet time = 170; physical activity = 50; school-related = 130; domestic/social = 60. Reference serves for dietary intake are: fruit and vegetables = 4; discretionary foods = 2, SSB = 1. The largest simulated decrease for SSB was − 1 serve to avoid negative values. *SSB* sugar sweetened beverages

Figure 1 provides an indication of how each behaviour is associated with physical functioning. Each individual plot shows the distribution of possible equivalent time-use (top row) and dietary (bottom row) combinations within 30 min of average time use (in 10-min increments) and 1 (sugar-sweetened beverages) or 1.5 (fruit and vegetables, discretionary foods) servings of the average dietary intake (in 0.5 serving increments). The left-most column shows equivalent behavioural differences for a + 0.5 unit difference in physical functioning scale. There are > 5700 equivalent options, the most obvious being lower discretionary food and sugar-sweetened beverage intake and higher fruit and vegetable intake, together with more physical activity and sleep. Moving to the right, the plots show that as the desired improvement in physical functioning scale becomes larger, there are fewer equivalent options available within the prescribed limits (30 min and 1/1.5 servings). For a + 2.5 difference in physical functioning, there are only 45 equivalent options (right-most column). Of these options, least flexibility is within dietary behaviours. At a physical functioning difference of + 2.5, all sugar-sweetened beverage options are reduced to the lower limit of hypothetical options at − 1 servings, and almost all discretionary food options are at − 1.5 serves, while all fruit vegetable options are increased to the highest possible amount at + 1.5 serves. There is more flexibility among time-use behaviours, but the only options available for physical activity are to increase it, preferably by 30 or 20 min, with only one option allowing just a 10-min increase of physical activity. Most of the sleep options involve increases in duration. The options for quiet time, screen time and self-care predominantly involve reductions of time spent in these behaviours.

Each row in Table 2 presents an equivalent behaviour change option associated with + 2.5 point difference on the physical functioning scale. Green/brown colouring represents less of a behaviour, while blue/pink represents more of a behaviour. There are clear patterns indicating that higher physical functioning scale is associated with the combination of higher physical activity, sleep, fruit and vegetables and lower screen time, self-care, quiet time, discretionary foods and sugar-sweetened beverages. However, there are some exceptions which suggest compromises are possible. For example, there is one option for reducing sleep by 10 min (row 1), but this must be accompanied by the best dietary options, and more quiet time, school-related activities and physical activity, along with less self-care, domestic/social and screen time. There appear to be trade-offs available between physical activity and sleep—equivalent options with higher sleep durations (+ 20 to + 30 min) tend to require lower increases in physical activity duration (only + 10 to + 20 min).

**Table 2:**
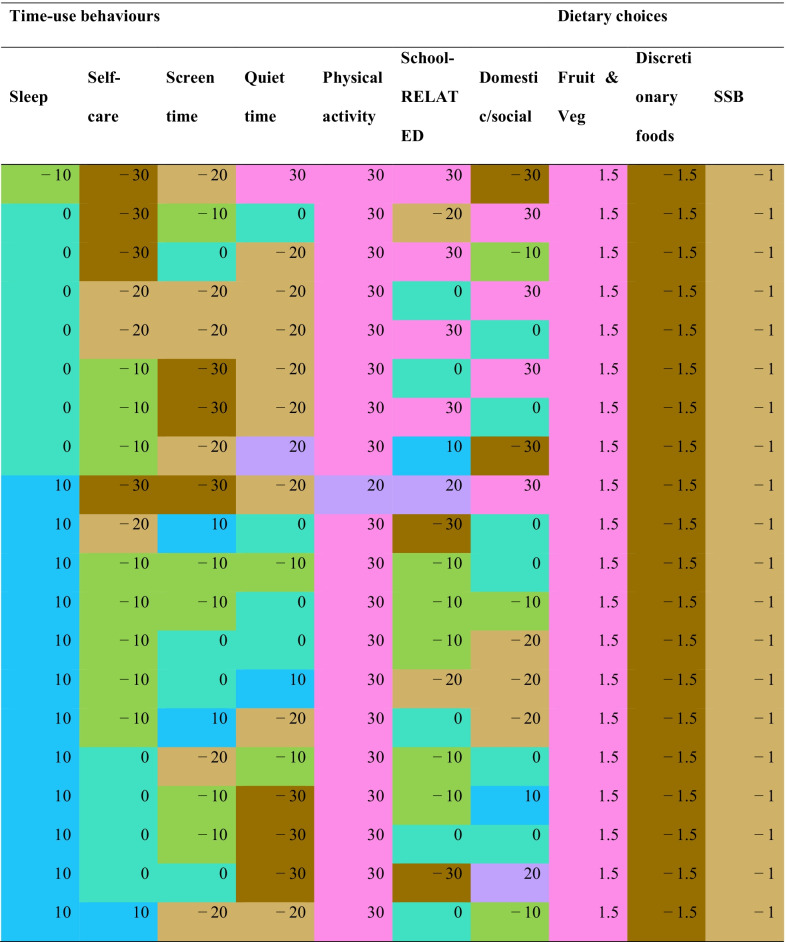

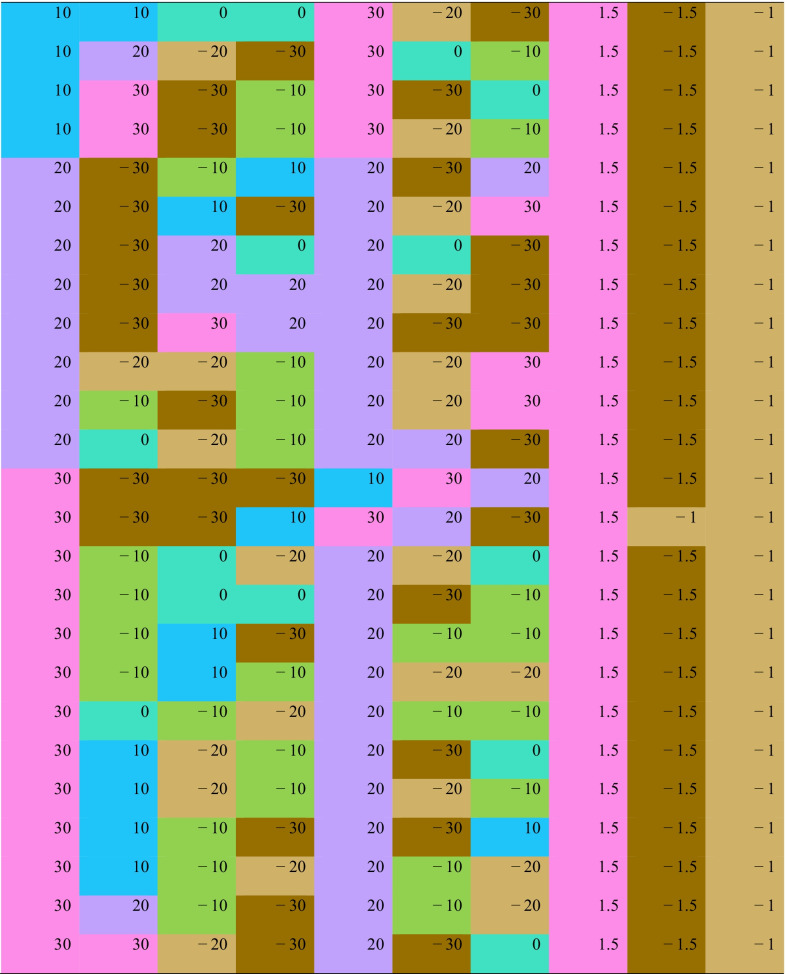
Equivalent behaviour change options associated with + 2.5 point difference in physical functioning scale (Teen PedsQL)

Analyses adjusted for age, sex and household socioeconomic status. Reference durations (min/day) for time-use behaviours are: sleep = 740; self care = 130; screen time = 160; quiet time = 170; physical activity = 50; school-related = 130; domestic/social = 60. Reference amounts (serves/day) for dietary intake are: fruit and vegetables = 4; discretionary foods = 2, SSB = 1. Green/brown colouring represents less of a behaviour, while blue/pink represents more of a behaviour. Colour-coding as for Fig. 1

*SSB* sugar-sweetened beverages

**Fig. 2:**
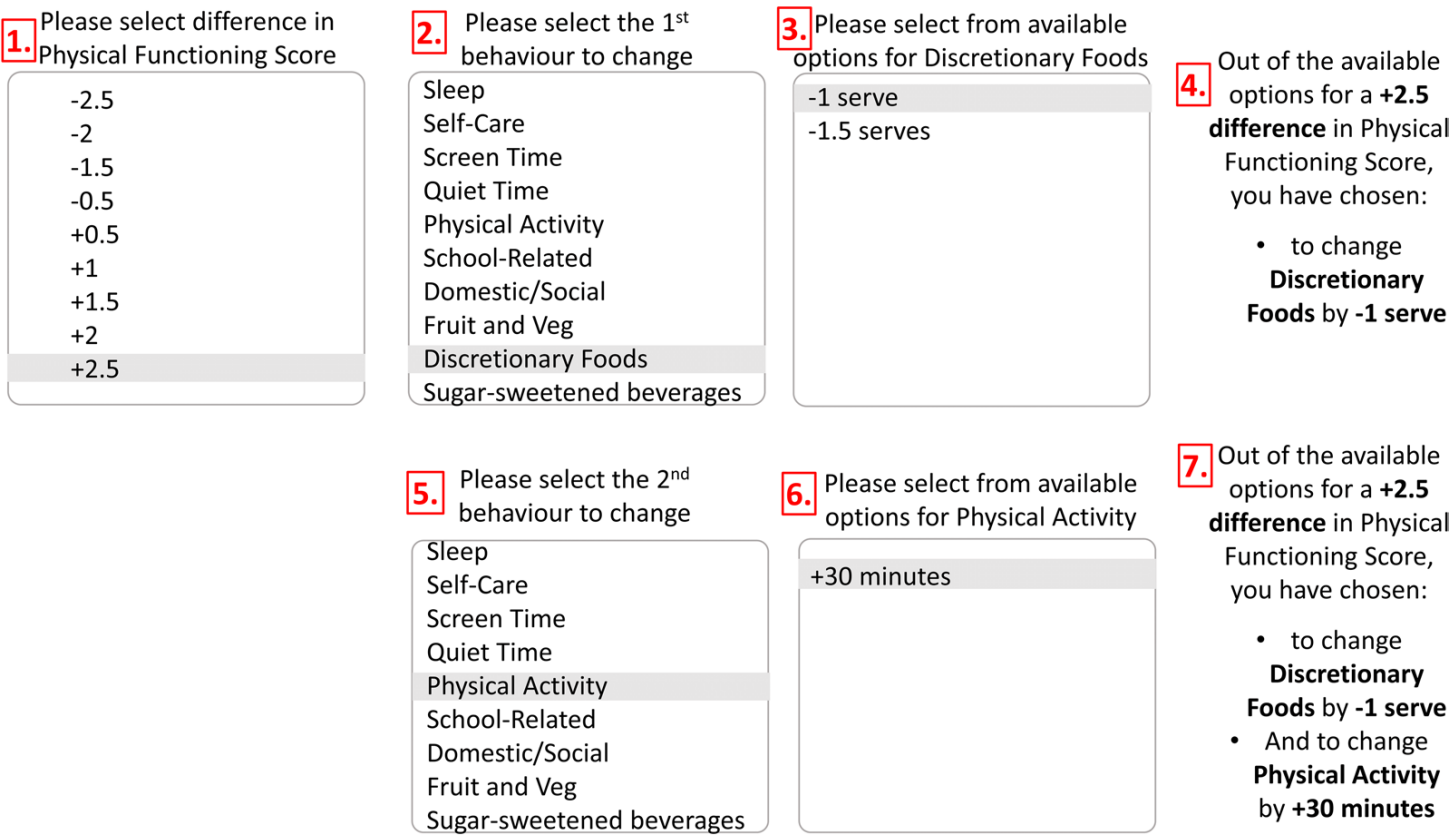
Mock-up of ShinyApp interface to allow users to explore equivalent behaviour-change options

Equivalent behaviour change options for physical functioning can be accessed using the ShinyApp at the following weblink: https://tystan.shinyapps.io/behaviourchange/ or downloaded from https://github.com/tystan/behaviourchange. The available options for difference in physical function range from − 2.5 to + 2.5, in 0.5-unit increments. Figure 2 is an impression of the app’s interface. The first step (Box 1) requires the app user to select their desired difference in physical functioning (+ 2.5 has been selected in the figure). Following this, Box 2 appears, requesting the app user to select the first behaviour they would like to change from the list of all available options (Discretionary Foods has been selected in the figure). Subsequently, they are presented with a list of available changes for the selected behaviour (Box 3). Figure 2 shows that for a + 2.5 difference in physical function, there are only two options available for Discretionary Foods (− 1 or − 1.5 serves). Once the user has selected the preferred amount of change for the first behaviour, they are presented with their selections (Box 4), before being prompted to select the second behaviour they would like to change (Box 5). The user continues to be prompted to select their next-preferred option from the remaining available behaviour-change options, until all behaviours are accounted for and they receive a summary of their personalised choices.

## Discussion

In a large sample of Australian adolescents, this study found cross-sectional evidence for equivalence among time-use and dietary behaviours for physical functioning. Within 30 min of the average adolescent’s time use and 1/1.5 servings of the average adolescents’ intake, we identified 45 equivalent options associated with + 2.5 physical functioning score (standardised effect size = 0.2). Compared to the average, all options associated with this difference in physical function had higher fruit and vegetable intake and physical activity, together with lower discretionary foods and sugar-sweetened beverage intake. Most options had higher sleep and lower self-care, screen time and quiet time.

Consistent with previous child studies exploring the equivalence of time-use behaviours in relation to health outcomes [7, 8], our study suggests sleep and physical activity could be traded off against each other. In other words, it appears that similar health gains are associated with either increasing sleep, or increasing physical activity, and that smaller increases are required if both sleep and physical activity are increased simultaneously. Previous studies only explored one type of time reallocation (one-for-remaining), in which an activity (e.g., sleep) is increased while all the remaining activities (sedentary time, light physical activity and moderate-to-vigorous physical activity) are decreased *pro rata* to compensate [7, 8]. The current study provides a more flexible approach that allows every possible time reallocation in 10-min increments to be explored, instead of imposing a particular reallocation pattern.

Our approach to consideration of time-use and dietary behaviours in terms of equivalency is novel, and consistent with how both physical activity and diet are increasingly targeted together in lifestyle interventions [15]. Systematic reviews and meta-analyses have reported better outcomes for combined exercise and diet interventions compared to single behaviour interventions on a range of outcomes (albeit mainly obesity) [28–31]. As both time use and diet have independently been linked with health [1, 32–34], it is not surprising that together they would have additive or synergistic effects on outcomes. Harnessing the potential for equivalency between time-use and dietary behaviours may allow lifestyle interventions to be tailored to the preferences and needs of the individual. If our cross-sectional findings hold true in longitudinal and intervention studies, an individual who wishes to maintain their current diet (perhaps for enjoyment, financial or cultural reasons) may obtain an equivalent benefit by improving time use. For an individual who is constrained to maintain their current time use (perhaps due to commuting, school or family commitments), improving diet could bring about the same reward. An individual choosing to improve both time-use and dietary behaviour may require smaller modifications to achieve an equivalent benefit.

There is some evidence that providing personal responsibility and choice in how to structure one’s behaviour change strategy may be the key to long term adherence and success [35]. This may be explained by the behavioural theory of self-determination which underpins incremental goal-setting interventions such as “Small Steps”, which have been shown to be effective and feasible to reduce sitting time among older adults [36]. A challenge with implementing personalised lifestyle intervention based on equivalences is the translation of equivalent behavioural options obtained from complex analytical statistical models, to both practitioners and the general public. Interactive interfaces such as our ShinyApp, which allow the user to interrogate the available options through successive decision-making processes, could be a solution. Users could plan out their own “Small Steps” with the app, setting goals that are suited to their own personal behavioural preferences and external constraints (e.g. school/work commitments, budgetary limitations on food choices). Once a robust evidence base for time-use and diet equivalences is available, these interactive interfaces must be co-designed with identified end-users to ensure they are accessible, meaningful and effective.

This study’s strengths include the large population-based sample, novel statistical methods which allow all daily time-use behaviours to be assessed simultaneously with dietary behaviours, and an innovative knowledge translation tool. The limitations must be acknowledged. The potential of selection bias exists as only 60% of the LSAC Wave 6K-cohort was included in this study, however sociodemographic characteristics of the included sample were almost identical to the complete cohort [sample vs. cohort: 50 vs. 51% female; 14.4 (SD 0.5) vs. 14.4 (SD 0.5) years old; socioeconomic *z*-score 0.06 (SD 1.00) vs. 0.00 (SD 1.00)]. We have used cross-sectional data which preclude any inference of causation—the equivalent behaviour-change options presented are more accurately perceived as equivalent lifestyles. All time-use, diet and health measures used in the analyses were self-reported by the adolescent participants, meaning there is potential for recall (participants may not accurately remember their behaviours) and social desirability (participants may respond in a way which makes their behaviours seem better—e.g. by reporting less screen time than they actually accumulated) bias. Each participant only contributed one time-use diary and recalled diet on the previous day only, thus these measures may not have captured their habitual behaviours. A mixture of school and non-school days was recalled, which we did not account for in the analysis. However, adding day-type (school/non-school) as an additional covariate did not change the statistical significance of the behavioural variables. The dietary behaviours considered in this study were limited to three food groups (fruit and vegetables, discretionary foods and sugar-sweetened beverages) and therefore did not represent the full 24-h dietary composition. The dietary measurement was limited to how often a food was consumed the previous day, with a ceiling of two or more instances. To improve interpretability, we assumed each instance of food intake was equivalent to one serve. As a result, we may have underestimated the total serves of foods consumed. The statistical model assumed a linear relationship between dietary variables and the outcome, meaning the interpretation of coefficients could be extrapolated to negative servings or impossibly high servings of foods. To address this, we constrained the model-based estimates to be within feasible and non-negative boundaries (within 1 or 1.5 servings of the mean). The simulated behaviour changes all originated from the means observed in the sample and are therefore suited for the “average” young person, but it is likely that different behaviour changes are required if the behaviour changes originated from different starting points. For example, to achieve the same absolute difference in health, a very inactive individual may require smaller increases in physical activity than a very active individual.

## Conclusion

Time-use and diet behaviours are both linked to young people’s physical functioning, and equivalent differences in physical functioning are associated with a range of behaviour-change options across some or all of these behaviours. Thus, young people may be able to choose an intervention strategy that is most desirable to them without loss of health benefit.

## Supplementary Information

**Additional file 1.** Time-use diary processing: Longitudinal Study of Australian Children, K cohort, Wave 6. The additional file provides further details on how the time-use diary activities were aggregated into the time-use domains used in this study.

## Data Availability

The data that support the findings of this study are available from the National Centre for Longitudinal Data (NCLD) at https://growingupinaustralia.gov.au/data-and-documentation/accessing-lsac-data but restrictions apply to the availability of these data, which were used under license for the current study, and so are not publicly available. Data are however available from the authors upon reasonable request and with permission of the NCLD.

## Abbreviations

zBMI: Body mass index z-score
MVPA: Moderate-to-vigorous physical activity
LSAC: Longitudinal Study of Australian Children
PedsQL: Pediatrics Quality of Life
SEP: Socioeconomic position
SSB: Sugar-sweetened beverages
