# Supplementary material for: Many different roads lead to Rome: equivalence of time-use for activity, sedentary and sleep behaviours and dietary intake profiles among adolescents
Source: J Act Sedentary Sleep Behav. 2022 Nov 1;1:6. doi: 10.1186/s44167-022-00005-1 (PMC11934514; doi:10.1186/s44167-022-00005-1)
Supplement: Supplementary file 1 — Additional file 1. Time-use diary processing: Longitudinal Study of Australian Children, K cohort, Wave 6. The additional file provides further details on how the time-use diary activities were aggregated into the time-use domains used in this study. [file 44167_2022_5_MOESM1_ESM.pdf]

Additional File 1. Time-use diary processing: Longitudinal Study of Australian Children, K cohort, Wave 6

Participants were mailed a paper-based time-use diary, and asked to complete it on the day before their scheduled home interview, using a pen containing a digital clock. They were asked to write down each of their daily activities and to use the clock in the pen to write down the time. They were prompted to fill separate text boxes with the time they work up, went to bed and went to sleep. If they had something to eat or drink during activities, they were instructed to place a sticker next to the corresponding activity.

Trained interviewers coded the activities recorded by the children into the individual activity classifications listed in Table 1. We collapsed these individual activities into activity macrodomains, which were further collapsed into the seven activities used in the analyses.

Table 1. Hierarchy of activities

**Activities used for analysis**	**Activity macrodomains**	**Individual activity classifications**
Physical activity	Sport	Archery, Shooting sports (individual or unstructured)<br>Athletics, Gymnastics (individual or unstructured)<br>Ball Sports (individual or unstructured)<br>Fitness, Gym, Exercise (individual or unstructured)<br>Martial arts, Dancing (individual or unstructured)<br>Motor Sports, Roller Sports, Cycling (individual or unstructured)<br>Organised individual sport and training<br>Organised team sports and training<br>Umpiring work<br>Water Ice Snow Sports (individual or unstructured)
Physical activity	Active Transport	By bike, scooter, skateboard etc<br>By foot
Physical activity	Play	Unstructured active play<br>Unstructured non-active play<br>Active club activities<br>Active activities
School-related	School-related	Arts<br>Attend courses excluding school/university<br>Clerical, office<br>Filling out the diary<br>Handwork crafts excl clothes making<br>School lessons
School-related	Study	Doing home work (via electronic device or not via electronic device)<br>Hobbies, collections<br>Playing musical instruments or singing for leisure

		Private music lessons/practice, academic tutoring<br>Reading or being read to for leisure
Domestic/Social	Work	Hospitality including fast food<br>Labourers and related workers<br>Volunteering work<br>Work Other<br>Working in a family business or farm<br>Apprenticeships trades persons<br>Retailing
Domestic/Social	Social	Attendance at concert/theatre<br>Attendance at museum, exhibition, art gallery<br>Attendance at other mass events<br>Attendance at zoo/ animal park/ botanic garden<br>Attending live sporting events<br>Chess, card, paper and board games, crosswords<br>Clubs<br>Communication<br>Dentist, Orthodontist, Doctor<br>Games of chance, gambling<br>Going out<br>Medical/ Health care<br>Negative face to face communication<br>Non-verbal interaction<br>Physiotherapist, Chiropractor<br>Talking on a landline phone<br>Talking on a mobile phone<br>Texting<br>Emailing<br>Weddings, funerals, rites of passage
Domestic/Social	Chores	Animal care<br>Babysitting<br>Car washing work<br>Car, boat, bike care<br>Chores<br>Cleaning grounds, garage, shed, outside of house chores<br>Cleaning, tidying<br>Clothes making<br>Design, Home Improvement<br>Food drink clean up<br>Food drink preparation<br>Gardening, maintenance chores<br>Gardening, lawn mowing<br>Heat, water, power upkeep<br>Home maintenance<br>Household management<br>Laundry, clothes care

		Packing<br>Pool care chores<br>Purchasing administrative services<br>Purchasing consumer goods<br>Purchasing other services<br>Purchasing personal care services<br>Purchasing durable goods<br>Purchasing repair services<br>Rubbish Recycling<br>Selling, disposing of household assets<br>Shopping<br>Taking care of siblings<br>Walking pets, playing with pets<br>Window shopping
Self Care	Eating	Eating/ Drinking
Self Care	Grooming	Cleaning teeth<br>Getting dressed, getting ready<br>Personal care<br>Medical Health Care<br>Showering/ bathing
Screen Time	Computer	Creating maintaining websites<br>Downloading posting media<br>Electronic device use<br>General application use<br>General Internet browsing<br>Internet shopping<br>Online chatting<br>Instant messaging<br>Spending time on social networking sites<br>Video chatting
Screen Time	TV	Watching TV programs or movies/videos
Screen Time	Videogame	Playing games, Electronic device
Quiet Time	Chill	Doing nothing<br>Listening to music<br>Non active activities<br>Talking face to face<br>Attendance at movies cinema<br>Time in bed not sleeping
Quiet Time	Passive Transport	By private motor vehicle/bike<br>By public chartered transport<br>Travel
Quiet Time	Spiritual	Religious activities ritual ceremonies<br>Religious groups<br>Religious practice
Sleep	Sleep	Sleep<br>Sleeping/napping (not end of day bed time)

3074 participants provided a time-use diary. These diaries underwent further processing to remove invalid data (Table 2).

Table 2: Time-Use Diary Processing Steps

Check	Rationale and Decision
Recorded duration of waking activities	Exclude if less than 10 h of waking activity are recorded (loss of 135 participants)
Is bed time recorded?	Only missing for 1 participant, who was watching TV starting at 11:30pm, thus not having recorded a bed time before midnight is reasonable and participant will not be excluded.
Is wake-up time recorded?	No missing wake up times
Are any activities recorded before wake up time?	Yes, n=89. Manual inspection of diaries shows that they have recorded activities shortly after midnight before going to bed later early that morning. Retain in analysis.
Are any activities recorded to start at the same time as wake up time?	Yes, n=590. This will cause the activity to be given zero duration, as the duration of activities is derived from subtracting the time-stamp of the former from the latter. Solution: If an activity start time is identical to wake-up time, alter wake-up time to be 1-second earlier than the activity. This way, 1 second will be allocated to "wake up", which will disappear when activity durations are later rounded to the nearest minute.
Are any activities recorded as starting after bed time?	Yes, n=900. Of these, 172 are due to bed time being early in that morning. Retain. Inspecting diaries shows that the remaining n=728 have recorded feasible after bed-time activities, such as reading, texting and playing computer games. Retain.
Are any activities recorded as starting after a p.m. go-to-sleep time?	Only in n=11 cases where go-to-sleep time was after 12 noon, and the activities do not seem reasonable, e.g., "packing a bag". Solution: any activities starting after go-to-sleep time will be removed, and only sleep retained.
How were eating/drinking episodes handled?	When eating and drinking start times were inserted during an activity, that eating and drinking episode appeared to continue until the start of the next activity. This resulted in excessively long eating and drinking durations, as the episode of eating/drinking would not appear to have finished until the next activity started. Solution: Any activities classified as "drinking" were capped at 5 minute, and "eating" at 15 minutes. Any time void was filled with the preceding activity.

Further processing of time-use data was undertaken prior to analysis:

Of n=2923 participants retained to this point, n=544 accumulated no time in Eating. These were considered invalid and excluded.

Of these n=2379 participants, n=198 recorded some time in "Don't know", thus did not have full 1440 min of activities. These were excluded.

Of these n=2181, n=15 recorded > 800 min sleep which was considered invalid and excluded.

A total of 2166 time-use diaries were retained. The seven activity variables were checked for zero values (Table 3), and these zeros were replaced with small values using a log-ratio EM algorithm.

Table 3. Zeros in activity variables

Activity	Number of non-zero values	Number of Zeros
Physical Activity	1605	561
School-related	1608	558
Domestic social	1746	420
Self Care	2166	0
Screen Time	1940	226
Quiet Time	2161	5
Sleep	2166	0
